# Eight cobweb spider species from China building detritus-based, bell-shaped retreats (Araneae, Theridiidae)

**DOI:** 10.3897/zookeys.1055.67620

**Published:** 2021-08-09

**Authors:** Zichang Li, Ingi Agnarsson, Yu Peng, Jie Liu

**Affiliations:** 1 The State Key Laboratory of Biocatalysis and Enzyme Engineering of China, College of Life Science, Hubei University, Wuhan 430062, Hubei, China Hubei University Wuhan China; 2 Faculty of Resources and Environmental Sciences, Hubei University, Wuhan 430062, Hubei, China University of Vermont Burlington United States of America; 3 School of Nuclear Technology and Chemistry & Biology, Hubei University of Science and Technology, Xianning 437100, Hubei, China Hubei University Wuhan China; 4 Department of Biology, University of Vermont, Burlington, VT, USA University of Vermont Burlington United States of America

**Keywords:** Bell-shaped retreat, *Campanicola*, new species, taxonomy, theridiid spiders, trash-decorating behaviour

## Abstract

Eight cobweb spider species building a detritus-based, bell-shaped retreat from China are reported in the current paper, including five new *Campanicola* species and three known species: *Campanicolaanguilliformis* Li & Liu, **sp. nov.**, *Campanicolafalciformis* Li & Liu, **sp. nov.**, *Campanicolaheteroidea* Li & Liu, **sp. nov.**, *Campanicolatauricornis* Li & Liu, **sp. nov.**, *Campanicolavolubilis* Li & Liu, **sp. nov.**, *Campanicolacampanulata* (Chen, 1993), *Campanicolaferrumequina* (Bösenberg & Strand, 1906), and *Parasteatodaducta* (Zhu, 1998). Among them, the male of *Parasteatodaducta* (Zhu, 1998) is described for the first time. We provide photographs of all species and descriptions for new species in the current paper. The type of bell-shaped retreat is rare in theridiid, and found only in four related genera. A natural next step upon completing this taxonomic study would be to analyse and understand the evolution of the retreat and related traits.

## Introduction

Orb-weaving spiders often employ a variety of ‘trash’ materials such as soil particles, plant detritus, prey remains and egg sacs into webs, which can effectively deceive predators, thereby increasing their chances of survival (Ma et al. 2020). This trash-decorating behaviour has mainly been reported in a few taxa, such as *Cyclosa* spiders (Herberstein et al. 2000), diguetid spiders (Gertsch 1958), the spider *Aziliavachoni* (Sewlal 2016) and cobweb spiders (Yoshida 2015). Among of them, some cobweb spiders are famous for building a bell-shaped retreat (Fig. 15), including four genera: *Achaearanea* Strand, 1929: *Achaearaneaglobispira* Henschel & Jocqué, 1994; *Cryptachaea* Archer, 1946: *Cryptachaeariparia* (Blackwall, 1834), *Cryptachaeaogatai* Yoshida, 2016; *Parasteatoda* Archer, 1946: *Parasteatodaducta* (Zhu, 1998), *Parasteatodaangulithorax* (Bösenberg & Strand, 1906), *Parasteatodatabulata* (Levi, 1980); *Campanicola* Yoshida, 2015: *Campanicolacampanulata* (Chen, 1993), *Campanicolaferrumequina* (Bösenberg & Strand, 1906), *Campanicolatanakai* Yoshida, 2015, *Campanicolachitouensis* Yoshida, 2015, *Campanicolaformosana* Yoshida, 2015 (Henschel and Jocqué 1994; Zhu 1998; Yoshida 2009, 2015, 2016).

In the past three years, a series of surveys on Chinese theridiid spiders were conducted by colleagues of Hubei University in China and yielded numerous new species. This is our first paper on Chinese cobweb spiders with the aim to describe the species building bell-shaped retreats including seven *Campanicola* species and one *Parasteatoda* species.

## Materials and methods

All specimens were kept in 100% ethanol and examined with an Olympus SZX16 stereomicroscope. Further details were studied under an Olympus BX51 compound microscope. Male and female genitalia were examined and illustrated after dissection. Epigynes were cleaned by Proteinase K solution. Male left palps and female epigynes were imaged with an Olympus BX51 compound microscope in ethanol and in Arabic gum. For SEM, specimens were treated according to Agnarsson (2004). Male palps were cleaned ultrasonically for 1 minute, transferred to 100% ethanol overnight, then air-dried. Palps were glued to round-headed rivets using conductive adhesive, then sputter coated and imaged by a field emission scanning electron microscope (FE-SEM JSM7100F, JEOL, JP). Spider body photos were obtained using a Leica 205C digital microscope. All images were assembled using Helicon Focus image stacking software. All measurements were obtained using a Leica 205C digital microscope and given in millimeters. Eye diameters were taken at the widest point. Leg lengths are given as: total length (femur, patella, tibia, metatarsus, tarsus). All specimens studied are deposited in Centre for Behavioural Ecology and Evolution (**CBEE**), College of Life Science, Hubei University, Wuhan, China.

### Abbreviations

**ALE** anterior lateral eyes

**AME** anterior median eyes

**C** conductor

**CD** copulatory duct

**Chd** cymbial hood

**CP** copulatory pore

**Cy** cymbium

**E** embolus

**FD** fertilization duct

**PLE** posterior lateral eyes

**PME** posterior median eyes

**S** spermathecae

**ST** subtegulum

**T** tegulum

**Ti** tibia

**I, II, III, IV** legs I to IV

## Taxonomy

### Family Theridiidae Sundevall, 1833

#### Genus Campanicola

Taxon classificationAnimaliaAraneaeTheridiidae

Yoshida, 2015

32209347-D98D-54B4-9B16-0180CFC5096D

##### Diagnosis.

*Campanicola* is similar to some *Achaearanea*, *Cryptachaea*, *Parasteatoda* in building a detritus-based and bell-shaped retreat, but can be distinguished morphologically from *Achaearanea* by the cymbium with cymbial hood and without a distal projection in *Campanicola*, but with a cymbial hook and large distal projections in *Achaearanea*; from *Cryptachaea* by the cymbium not extending beyond the alveolus, the tegulum depressed and the copulatory ducts long in *Campanicola*, but the cymbium extending beyond alveolus, the tegulum spherical and the copulatory ducts short in *Cryptachaea*; from *Parasteatoda* by the conductor tip curved dorsally, the embolus and the copulatory ducts thin and the atrium small in *Campanicola*, but the conductor tip curved ventrally, the embolus and the copulatory ducts thick, the atrium large in *Parasteatoda* (Yoshida 2008, 2015; Vanuytven 2021).

#### Campanicola campanulata

Taxon classificationAnimaliaAraneaeTheridiidae

(Chen, 1993)

4059AB14-F1E7-5B99-8749-C96005582276

**Figure 1.**
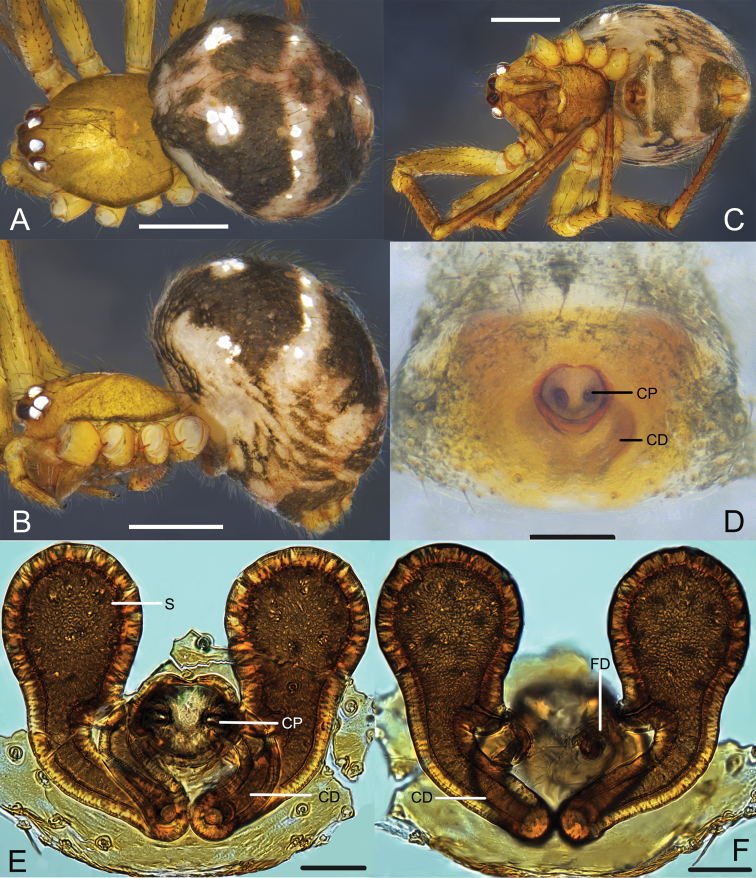
*Campanicolacampanulata* (Chen, 1993) **A–C** female habitus (**A** dorsal view **B** lateral view **C** ventral view) **D–F** female epigynum (**D** ventral view, uncleared **E** ventral view, cleared and embedded in Arabic gum **F** dorsal view, cleared and embedded in Arabic gum). Scale bars: 0.5 mm (**A–C**); 0.1 mm (**D–F**).

**Figure 2.**
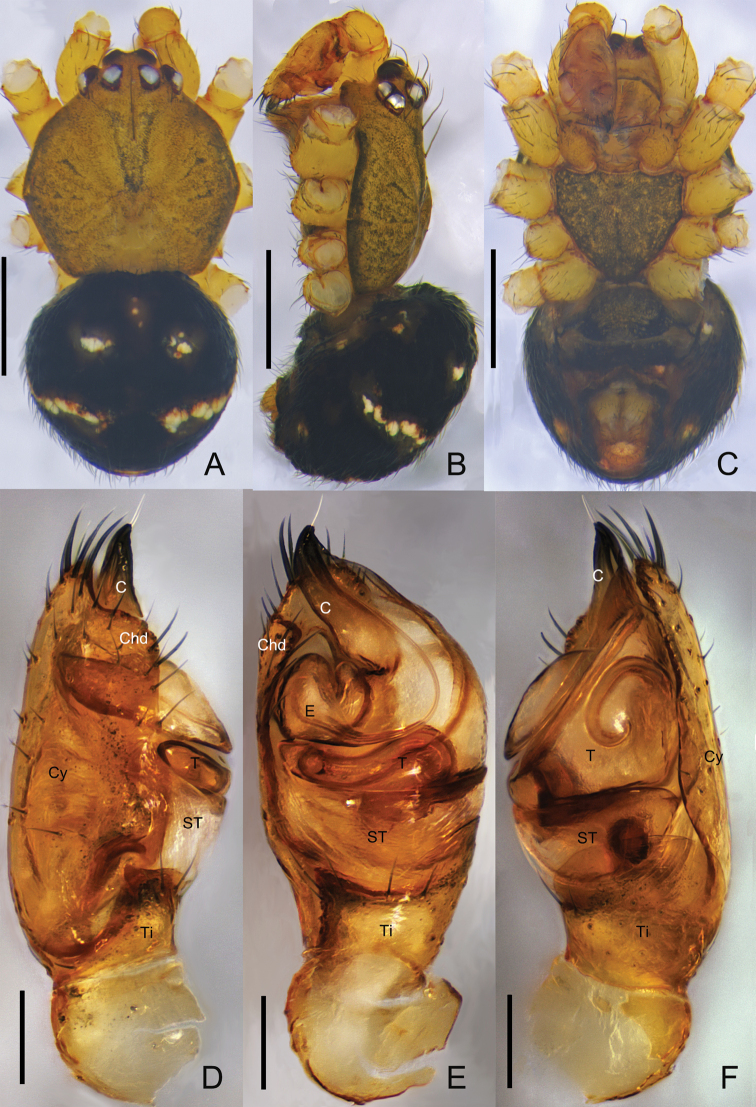
*Campanicolacampanulata* (Chen, 1993) **A–C** male habitus (**A** dorsal view **B** lateral view **C** ventral view) **D–F** male left palp (**D** prolateral view **E** ventral view **F** retrolateral view). Scale bars: 0.5 mm (**A–C**); 0.1 mm (**D–F**).

**Figure 3.**
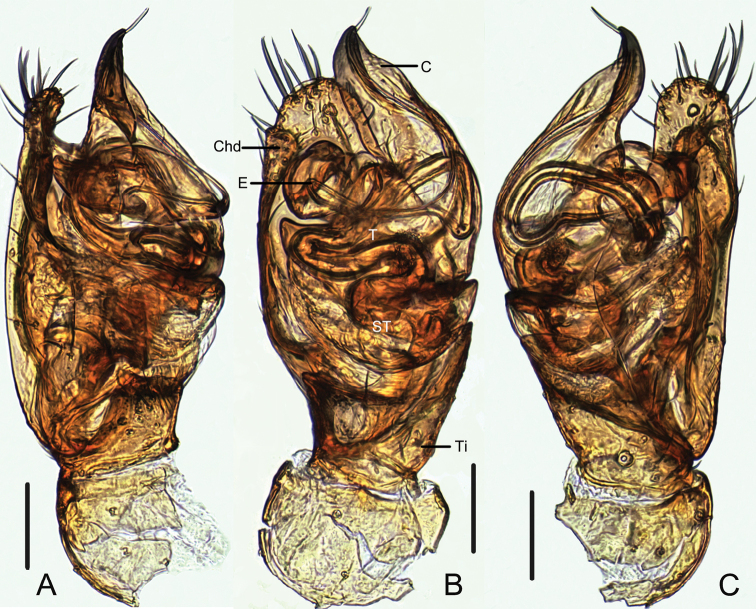
*Campanicolacampanulata* (Chen, 1993) male left palp embedded in Arabic gum (**A** prolateral view **B** ventral view **C** retrolateral view). Scale bars: 0.1 mm.

Figs 1 , 2 , 3 , 15 , 16

Achaearanea campanulata Chen, 1993: 36, f. 1–5 (description of male and female).
Achaearanea campanulata : Chen and Zhang 1991: 138, f. 130.1–5 (male and female; *nomen nudum*); Zhu 1998: 88, f. 50A–E (male and female); Song, Zhu and Chen 1999: 86, f. 38E, F, M, N (male and female); Zhu and Zhang 2011: 71, f. 36A–E (male and female); Yin et al. 2012: 250, f. 80A–E (male and female).
Parasteatoda campanulata : Yoshida 2008: 39 (male and female transferred from Achaearanea).
Campanicola campanulata : Yoshida 2015: 33 (male and female transferred from Parasteatoda).

##### Material examined.

**China, Guizhou Province**: 1 ♂, 13 ♀, Suiyang County, Wenquan Town, Shuanghe Village, Shanwang Cave (27°57'56"N, 107°9'26"E, 756 m), 8–10 May 2018, F.X. Liu, W. Ding and Z.C. Li leg.; 1 ♂, 3 ♀, Suiyang County, Wenquan Town, Dishui Village, Manwang Cave and village road (28°14'30"N, 107°0'56"E, 650 m), 11 May 2018, F.X. Liu, W. Ding and Z.C. Li leg.; 1 ♂, 6 ♀, Fuquan City, Kouhuang Cave (26°33'31"N, 107°12'55"E, 1030 m), 13 May 2018, F.X. Liu, W. Ding and Z.C. Li leg.; **Hunan Province**: 15 ♀, Sangzhi County, Tianpingshan Forest Farm (29°47'N, 110°5'12"E, 1359 m), 1–3 June 2018, F.X. Liu, J.S. Lu, J.C. Zhang, R. Zhong and Z.C. Li leg.; 11 ♀, Sangzhi County, Meijiashan Park (29°24'24"N, 110°9'28"E, 336 m), 4 June 2018, F.X. Liu, J.S. Lu, J.C. Zhang, R. Zhong and Z.C. Li leg.; 2 ♀, Zhangjiajie City, Zhangjiajie National Forest Park, footpath of The Yellow Rock Village and The Golden Whip Brook (29°19'22"N, 110°25'38"E, 452–780 m), 6 June 2018, F.X. Liu, J.S. Lu, J.C. Zhang, R. Zhong and Z.C. Li leg.; 1 ♀, Changsha City, Yuelu Mountain (28°11'33"N, 112°56'6"E, 210 m), 12 August 2018, Z.W. Deng and Z.C. Li leg.; 1 ♀, Hengyang City, Hengshan scenic spot, Fanyin Valley (27°16'22"N, 112°42'41"E, 410 m), 17 August 2018, Z.W. Deng and Z.C. Li leg.; **Sichuan Province**: 8 ♀, Emeishan City, Emei Mountain Scenic Spot, footpath from Wuxian Gang to Wannian Temple (29°35'3"N, 103°22'55"E, 940 m), 21 September 2018, F.X. Liu, Z.W. Deng and Z.C. Li leg.; 17 ♀, Leshan City, Leshan Giant Buddha Scenic Spot (29°32'28"N, 103°46'19"E, 380 m), 24 September 2018, F.X. Liu, Z.W. Deng and Z.C. Li leg.; 1 ♀, Ya’an City, Baoxing County, Panda Square (30°22'10"N, 102°48'50"E, 1060 m), 27 September 2018, F.X. Liu, Z.W. Deng and Z.C. Li leg.; 5 ♀, Ya’an City, Baoxing County, Longmen Town (30°15'14"N, 103°1'20"E, 810 m), 28 September 2018, F.X. Liu, Z.W. Deng and Z.C. Li leg.; **Hubei Province**: 7 ♀, Lichuan City, Tenglongdong Scenic Spot (30°16'10"N, 108°56'15"E, 1070 m), 5 October 2018, F.X. Liu, Z.W. Deng and Z.C. Li leg.; 2 ♀, Jianshi County, Chaoyangguan (30°35'45"N, 109°42'52"E, 650 m), F.X. Liu, Z.W. Deng and Z.C. Li leg.

##### Diagnosis.

The females of *C.campanulata* differ from all other *Campanicola* species by the copulatory pores that are circular and not clinging to the atrium margin, and the copulatory ducts that curve only twice (Fig. 1D–F). Males differ from all other *Campanicola* species by the shorter and darker conductor tip (Fig. 2D–F).

##### Description.

See Zhu (1998).

##### Distribution.

China (Sichuan, new province record; Hubei, Hunan, Guizhou, Zhejiang, Fujian, Henan) (Fig. 16).

#### Campanicola ferrumequina

Taxon classificationAnimaliaAraneaeTheridiidae

(Bösenberg & Strand, 1906)

EB7D43CE-7BCF-5029-B4C6-53AB311903CB

**Figure 4.**
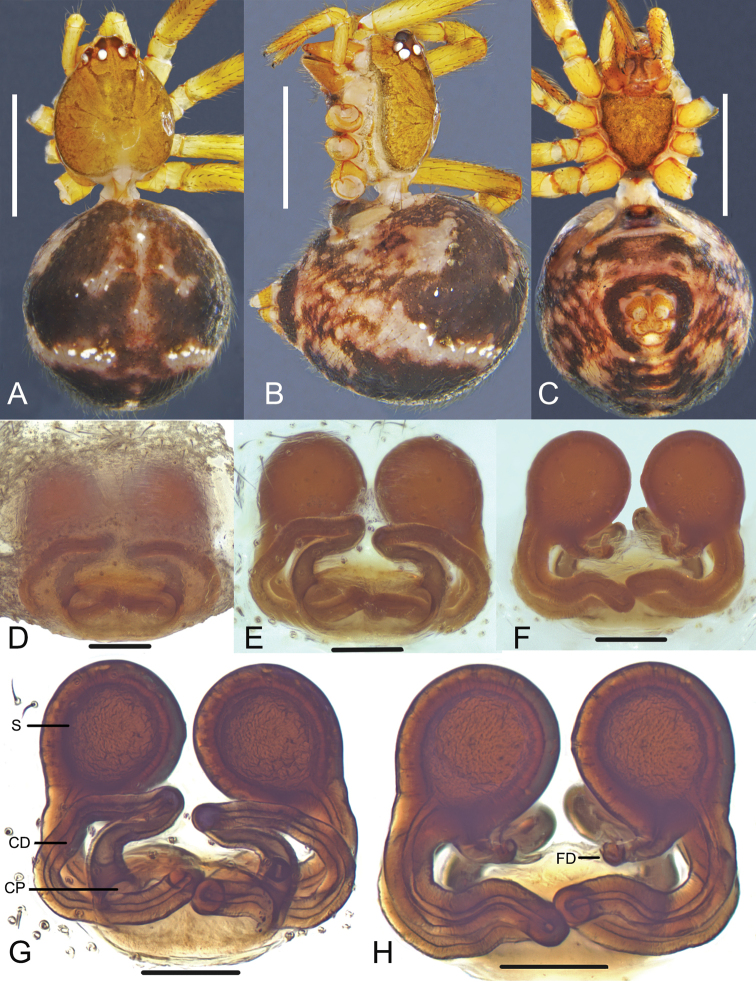
*Campanicolaferrumequina* (Bösenberg & Strand, 1906) **A–C** female habitus (**A** dorsal view **B** lateral view **C** ventral view) **D–H** female epigynum (**D** ventral view, uncleared **E** ventral view, cleared **F** dorsal view, cleared **G** ventral view, cleared and embedded in Arabic gum **H** dorsal view, cleared and embedded in Arabic gum). Scale bars: 0.5 mm (**A–C**); 0.1 mm (**D–H**).

**Figure 5.**
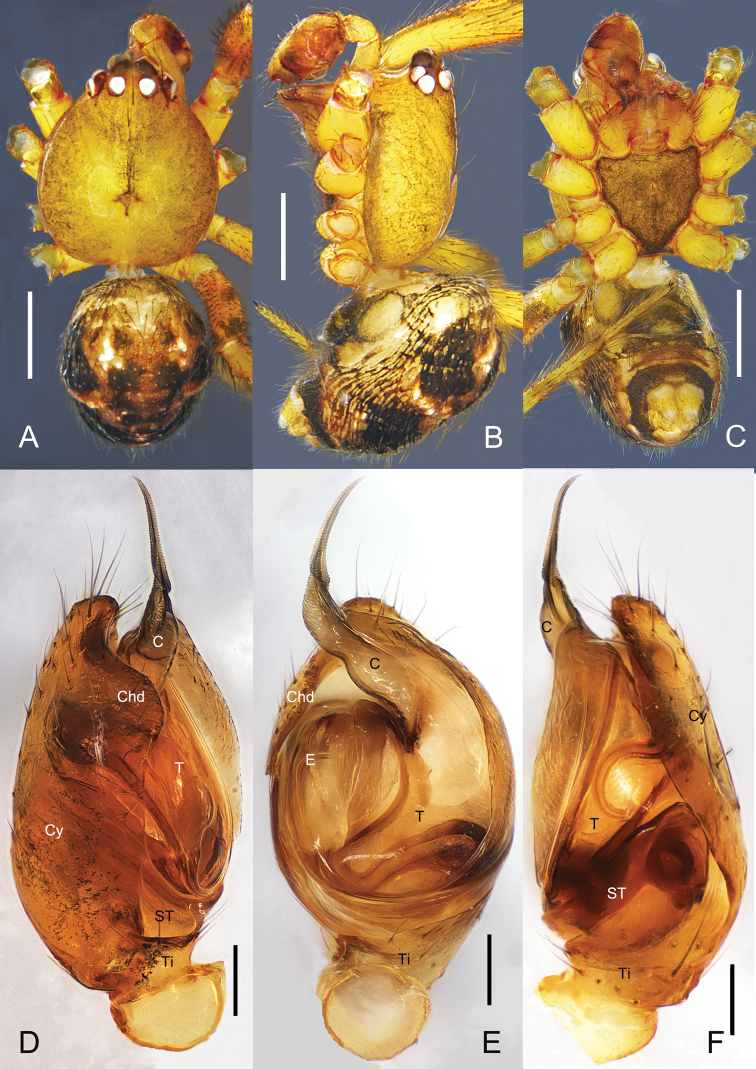
*Campanicolaferrumequina* (Bösenberg & Strand, 1906) **A–C** male habitus (**A** dorsal view **B** lateral view **C** ventral view) **D–F** male left palp (**D** prolateral view **E** ventral view **F** retrolateral view). Scale bars: 0.5 mm (**A–C**); 0.1 mm (**D–F**).

**Figure 6.**
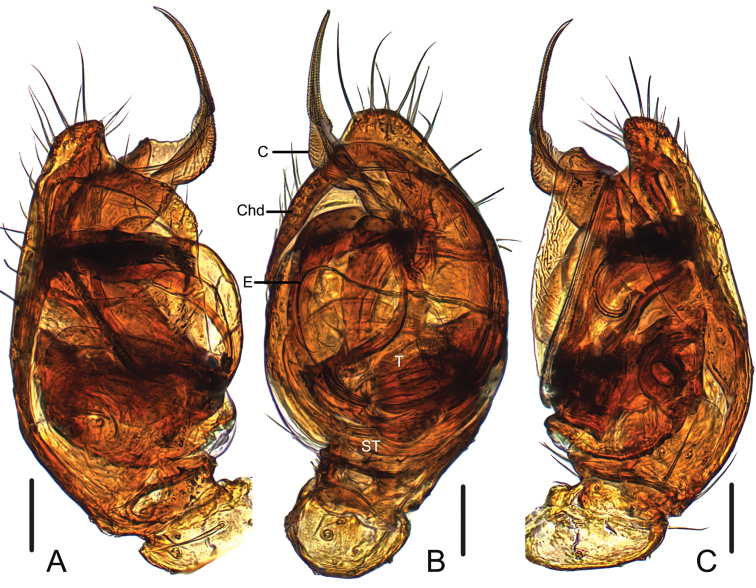
*Campanicolaferrumequina* (Bösenberg & Strand, 1906) male left palp embedded in Arabic gum (**A** prolateral view **B** ventral view **C** retrolateral view). Scale bars: 0.1 mm.

Figs 4 , 5 , 6 , 15 , 16

Theridion ferrum-equinum Bösenberg & Strand, 1906: 139, pl. 12, f. 261 (description of male and female); Oi 1957: 45, f. 1–3 (f); Yaginuma 1958: 70, f. 2, 7 (female).
Theridion meum Bösenberg & Strand, 1906: 145, pl. 12, f. 294 (description of female).
Theridion ferrumequinum : Saito 1959: 70, f. 65A–C (female); Yaginuma 1960: 36, f. 34.5 (female); Yaginuma 1971: 36, f. 34.5 (f); Ono 1981c: 2, f. 1 (female, synonymy).
Achaearanea ferrumequina : Yoshida 1983: 40 (male and female transferred from Theridion); Yaginuma 1986: 34, f. 19.8 (female); Chikuni 1989: 31, f. 5 (male and female); Zhu 1998: 99, f. 56A–C (female); Song, Zhu and Chen 1999: 90, f. 39G, H (female); Yoshida, 2000: 140, f. 4–7 (male and female); Namkung 2002: 85, f. 13.3A, B (male and female); Namkung 2003: 87, f. 13.3A, B (male and female); Yoshida 2003: 102, f. 250–253, 574 (male and female); Yin et al. 2012: 255, f. 84A–E (male and female).
Parasteatoda ferrumequina : Yoshida 2008: 39 (male and female transferred from Achaearanea); Yoshida 2009: 382, f. 258–259 (male and female).
Campanicola ferrumequina : Yoshida 2015: 33 (male and female transferred from Parasteatoda).

##### Material examined.

**China, Hunan Province**: 3 ♂, 32 ♀, Zhangjiajie City, Zhangjiajie National Forest Park, footpath of The Yellow Rock Village and The Golden Whip Brook (29°19'22"N, 110°25'38"E, 452–780 m), 6 June 2018, F.X. Liu, J.S. Lu, J.C. Zhang, R. Zhong and Z.C. Li leg.; **Fujian Province**: 1 ♂, 15 ♀, Wuyishan City, Wuyi Mountain Natural Reserves (26°39'42"N, 117°56'24"E, 399 m), 28–31 August 2019, Y. Zhong and F.J. Liu leg.

##### Diagnosis.

The females of *C.ferrumequina* differ from all other *Campanicola* species by the almost overlapping copulatory ducts except the short segment starting from the copulatory pores (Fig. 4F, H). Males differ from all other *Campanicola* species by the long conductor tip and the part of the conductor above the cymbium with helical lines (Figs 5D–F, 6A–C).

##### Description.

See Zhu (1998) and Yoshida (2000).

##### Distribution.

China (Hunan, Fujian, Sichuan, Hainan), Korea, Japan (Fig. 16).

#### Campanicola anguilliformis

Taxon classificationAnimaliaAraneaeTheridiidae

Li & Liu sp. nov.

203FE972-0E19-5650-B982-D529E2A86DBA

http://zoobank.org/989FD31C-343E-40E1-9063-E0ED7924B6DD

Figs 7 , 16

##### Type material.

***Holotype*: ♀, China, Fujian Province**: Wuyishan City, Wuyi Mountain Natural Reserves (26°39'42"N, 117°56'24"E, 399 m), 28 August 2019, Y. Zhong and F.J. Liu leg. **Paratypes**: 1 ♀, same data as holotype.

##### Diagnosis.

**Figure 7.**
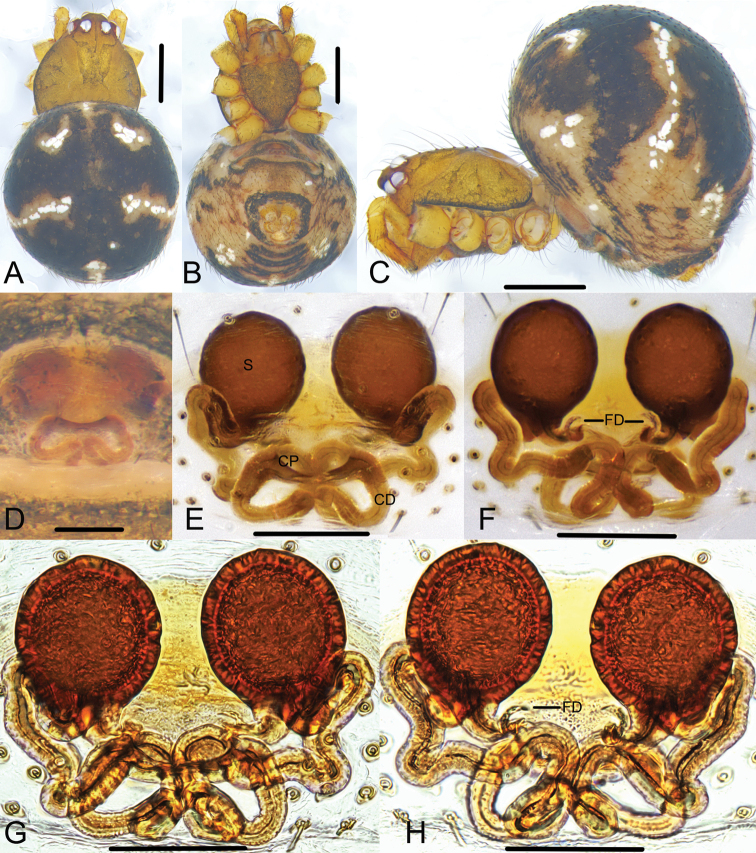
*Campanicolaanguilliformis* sp. nov. **A–C** female habitus (**A** dorsal view **B** ventral view **C** lateral view) **D–H** female epigynum (**D** ventral view, not dissected **E** ventral view, cleared **F** dorsal view, cleared **G** ventral view, cleared and embedded in Arabic gum **H** dorsal view, cleared and embedded in Arabic gum). Scale bars: 0.5 mm (**A–C**); 0.1 mm (**D–H**).

This new species is similar to *C.ferrumequina*, *C.tauricornis* sp. nov. and *C.volubilis* sp. nov. in having spherical spermathecae, and long and looped copulatory ducts, but can be distinguished from them by the direction of the copulatory duct: it starts from the middle and anterior part of the atrium, extends posteriorly, then curves several times and enters the spermathecae laterally and ventrally in *C.anguilliformis* sp. nov. (Fig. 7E–H); it starts from the lateral and anterior part of the atrium, extends anteriorly, then curves several times and enters the spermathecae laterally and dorsally in *C.ferrumequina* (Fig. 4E–H); it starts from the lateral and anterior part of the atrium, extends anteriorly, then curves several times and enters the spermathecae laterally and ventrally in *C.tauricornis* sp. nov. and *C.volubilis* sp. nov. (Figs 10D–G, 11D–G).

##### Etymology.

The specific name is derived from the Latin adjective *anguilliformis*, meaning eel-like, referring to the eel-like copulatory ducts; adjective.

##### Description.

**Male** unknown. **Female (holotype)**: Total length 2.06. Prosoma 0.91 long, 0.80 wide, brownish yellow, with black margin. Sternum 0.55 long, 0.51 wide, yellow, peltate, with sparse setae. Opisthosoma 1.45 long, 1.16 wide, dorsum black, with two pairs of transverse patterns in the shape of mustache and a longitudinal stripe, irregular white spots distributed in the patterns and the stripe; venter yellowish, with few bright white spots. Anal tubercle yellow. Spinnerets surrounded with black ring, without colulus (Fig. 7A–C). Diameters of eyes: AME 0.07, ALE 0.08, PME 0.09, PLE 0.07. Interdistances of eyes: AME-AME 0.06, AME-ALE 0.03, PME-PME 0.07, PME-PLE 0.05. Clypeus 0.16 high, yellow. Chelicerae yellow, promargin with 2 teeth. Endites yellow. Labium 0.19 long, 0.05 wide, yellow and rectangular, fused with sternum. Measurements of legs: I 3.58 (1.12, 0.29, 0.80, 0.89, 0.48), II 2.50 (0.77, 0.29, 0.47, 0.56, 0.41), III 1.90 (0.60, 0.22, 0.32, 0.42, 0.34), IV 3.00 (0.98, 0.32, 0.62, 0.66, 0.42). Leg formula: I-IV-II-III. Femur and patella light yellow, tibia, metatarsus and tarsus orange yellow. Femur, tibia and metatarsus with dark brown ring. Epigynum: atrium depression, small and oval; copulatory pores distinguishable, close to each other, located on the middle and anterior part of atrium; copulatory ducts long, winding, lightly sclerotized, connected with spermathecae from anterior-ventral part; spermathecae spherical; fertilization ducts short, curved and tapering (Fig. 7E, F).

##### Distribution.

China (Sichuan) (Fig. 16).

#### Campanicola falciformis

Taxon classificationAnimaliaAraneaeTheridiidae

Li & Liu sp. nov.

FCA4CDB6-10C2-5CF6-B059-142A9B5D0479

http://zoobank.org/F115BCB2-5F8E-405A-86F1-49B1CF49F8ED

Figs 8 , 16

##### Type material.

***Holotype* ♀, China, Sichuan Province**: Emeishan City, Emei Mountain Scenic Spot, footpath from Wuxian Gang to Wannian Temple (29°35'3"N, 103°22'55"E, 940 m), 21 September 2018, F.X. Liu, Z.W. Deng and Z.C. Li leg. **Paratypes**: 12 ♀, same data as holotype; **Sichuan Province**: 2 ♀, Leshan City, Leshan Giant Buddha Scenic Spot (29°32'28"N, 103°46'19"E, 380 m), 24 September 2018, F.X. Liu, Z.W. Deng and Z.C. Li leg.; 1 ♀, Ya’an City, Baoxing County, Fengtong Stronghold (30°34'21"N, 102°52'59"E, 1540 m), 26 September 2018, F.X. Liu, Z.W. Deng and Z.C. Li leg.; 3 ♀, Ya’an City, Baoxing County, Panda Square (30°22'10"N, 102°48'50"E, 1060 m), 27 September 2018, F.X. Liu, Z.W. Deng and Z.C. Li leg.; 1♀, Ya’an City, Baoxing County, Longmen Town (30°15'14"N, 103°1'20"E, 810 m), 28 September 2018, F.X. Liu, Z.W. Deng and Z.C. Li leg.

##### Diagnosis.

**Figure 8.**
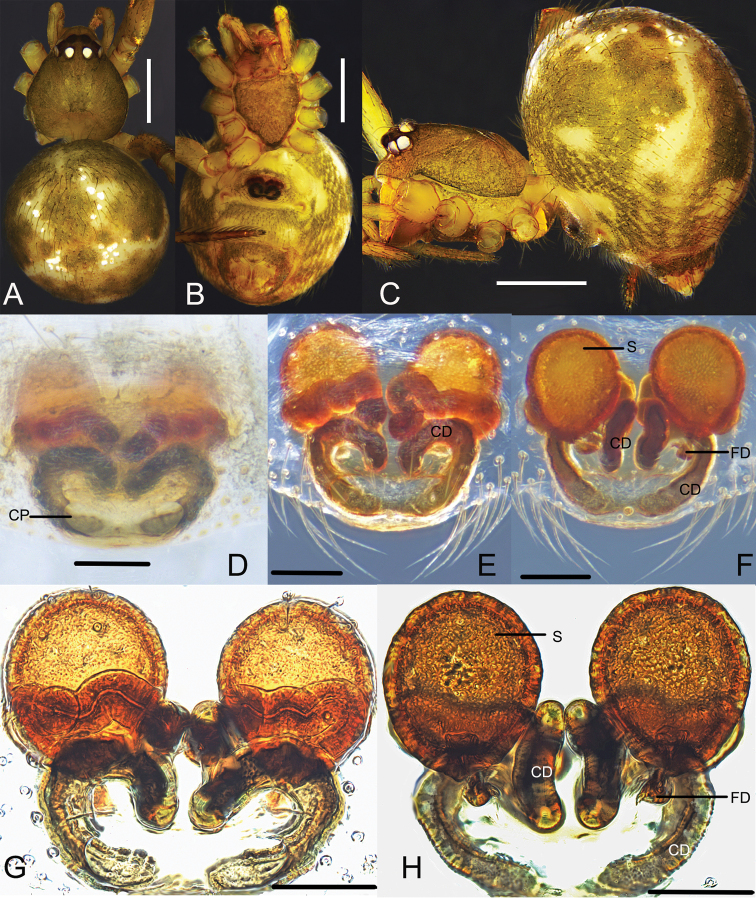
*Campanicolafalciformis* sp. nov. **A–C** female habitus (**A** dorsal view **B** ventral view **C** lateral view) **D–H** female epigynum (**D** ventral view, uncleared **E** ventral view, cleared **F** dorsal view, cleared **G** ventral view, cleared and embedded in Arabic gum **H** dorsal view, cleared and embedded in Arabic gum). Scale bars: 0.5 mm (**A–C**); 0.1 mm (**D–H**).

This new species can be distinguished from other *Campanicola* species, in the female, by the position of the copulatory pores: located on the lateral and posterior part of atrium in this new species while located anteriorly in other *Campanicola* species; and the relative position of the copulatory ducts to the spermathecae: partial copulatory ducts overlap the posterior-ventral half of the spermathacea in this new species (Fig. 8E, G), but nearly all copulatory ducts are below the spermathecae in other *Campanicola* species.

##### Etymology.

The specific name is derived from the Latin adjective *falciformis*, meaning doubly falcate, referring to the falcate copulatory ducts in ventral view; adjective.

##### Description.

**Male** unknown. **Female (holotype)**: Total length 2.29. Prosoma 0.92 long, 0.79 wide, brown, with dark brown margin. Sternum 0.49 long, 0.47 wide, yellow, peltate, with sparse setae. Opisthosoma 1.45 long, 1.38 wide, yellowish-brown, dorsum center with an angle bracket-shaped pale-yellow stripe either side of the midline, dorsum posterior with lateral pale-yellow stripe, and many bright white spots are distributed in the stripes (Fig. 8A). Anal tubercle yellow. Spinnerets surrounded with blackish brown ring, without colulus. Diameters of eyes: AME 0.06, ALE 0.09, PME 0.10, PLE 0.10. Interdistances of eyes: AME-AME 0.08, AME-ALE 0.03, PME-PME 0.08, PME-PLE 0.04. Clypeus 0.16 high, yellow. Chelicerae yellow, promargin with 2 teeth. Endites orange yellow. Labium 0.20 long, 0.06 wide, yellow and rectangular, fused with sternum. Measurements of legs: I 3.67 (1.17, 0.29, 0.79, 0.93, 0.49), II 2.52 (0.80, 0.25, 0.49, 0.58, 0.40), III 2.03 (0.62, 0.29, 0.35, 0.38, 0.39), IV 3.01 (1.01, 0.27, 0.65, 0.66, 0.42). Leg formula: I-IV-II-III. Femur, patella, tibia and metatarsus light yellow, tarsus yellow. Femur, tibia and metatarsus with dark brown ring. Epigynum: atrium depression and oval; copulatory pores apparent, widely separated with each other, located on the lateral and posterior part of atrium; copulatory ducts long and winding, the part of copulatory ducts near copulatory pores is more sclerotized than the part near spermathecae, the part near spermathecae overlaps half of the spermathecae, and connected with spermathecae from posterior and lateral part; spermathecae nearly spherical to oval; fertilization ducts short, tapering gradually (Fig. 8E, F).

##### Distribution.

China (Sichuan) (Fig. 16).

#### Campanicola heteroidea

Taxon classificationAnimaliaAraneaeTheridiidae

Li & Liu sp. nov.

88F724CE-088C-50DC-A3BE-154E0739CE69

http://zoobank.org/67E9B368-0AF8-4EFA-BD84-93584BFCAFF8

Figs 9 , 16

##### Type material.

***Holotype*: ♀, China, Guizhou Province**: Liupanshui City, Zhongshan District (26°35'50"N, 104°49'10"E, 1760 m), 26 August 2020, B. Liang and J.H. Wang leg.

##### Diagnosis.

**Figure 9.**
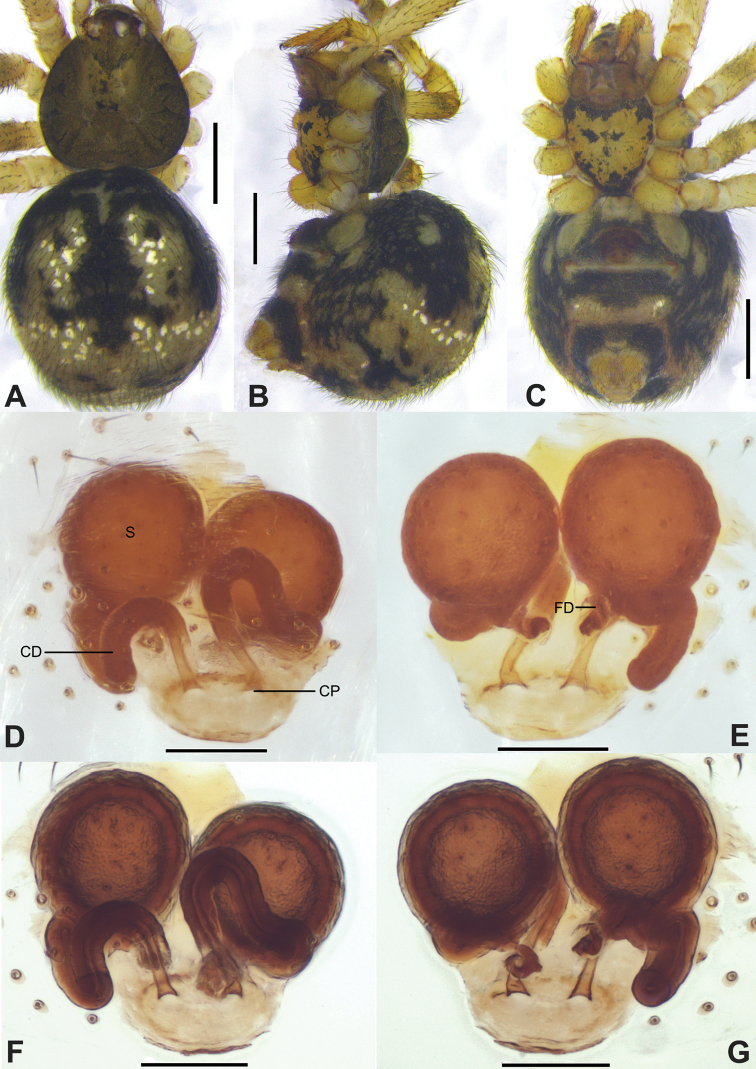
*Campanicolaheteroidea* sp. nov. **A–C** female habitus (**A** dorsal view **B** lateral view **C** ventral view) **D–G** female epigynum (**D** ventral view, cleared **E** dorsal view, cleared **F** ventral view, cleared and embedded in Arabic gum **G** dorsal view, cleared and embedded in Arabic gum). Scale bars: 0.5 mm (**A–C**); 0.1 mm (**D–G**).

This new species can be distinguished from other *Campanicola* species by the following characteristics: epigynum dissymmetric; copulatory pores located on the anterior margin of the atrium; copulatory ducts short, winding simply (Fig. 9D, E).

##### Etymology.

The specific name is derived from the Latin adjective *heteroideus*, meaning asymmetrical, referring in essence to the dissymmetric epigynum structure; adjective.

##### Description.

**Male** unknown. **Female (holotype)**: Total length 4.12. Prosoma 1.66 long, 1.54 wide, brown, with few black patches in the centre. Sternum 0.90 long, 0.93 wide, yellow, with black patches and sparse setae. Opisthosoma 2.45 long, 2.22 wide, oval, armed with black setae, dorsum yellowish with a middle longitudinal black belt which is surrounded with bright white spots, and more laterally mottled. Venter yellowish-brown, with a broad black transverse stripe next to epigastric furrow. Anal tubercle yellowish. Spinnerets yellow, surrounded with black ring, without colulus (Fig. 9A–C). Diameters of eyes: AME 0.07, ALE 0.06, PME 0.05, PLE 0.05. Interdistances of eyes: AME-AME 0.04, AME-ALE 0.05, PME-PME 0.05, PME-PLE 0.06. Clypeus 0.16 high, yellow, with black posterior margin. Chelicerae yellowish, promargin with 2 teeth. Endites brown. Labium 0.21 long, 0.09 wide, yellowish-brown, rectangular, fused with sternum. Measurements of legs: I 4.84 (1.53, 0.22, 1.18, 1.23, 0.68), II 3.16 (0.99, 0.26, 0.69, 0.67, 0.55), III 2.63 (0.82, 0.25, 0.50, 0.58, 0.48), IV 4.03 (1.35, 0.38, 0.80, 0.90, 0.60). Leg formula: I-IV-II-III. Legs yellowish, with dark brown ring. Epigynum: atrium depression and oval; copulatory pores pronounced, located on anterior margin of atrium; copulatory ducts short, winding simply, lightly sclerotized and dissymmetric, connected with spermathecae from anterior part; spermathecae spherical; fertilization ducts short and thin (Fig. 9D, E).

##### Distribution.

China (Guizhou) (Fig. 16).

#### Campanicola tauricornis

Taxon classificationAnimaliaAraneaeTheridiidae

Li & Liu sp. nov.

2A6EF14F-3CC1-52A5-A440-92EF7631B2E6

http://zoobank.org/DE68CCD7-7823-49F8-932D-14F7A2402C72

Figs 10 , 16

##### Type material.

***Holotype*: ♀, China, Hainan Province**: Limushan National Forest Park (19°10'N, 109°39'E), 20 July 2020, J. Liu leg.

##### Diagnosis.

**Figure 10.**
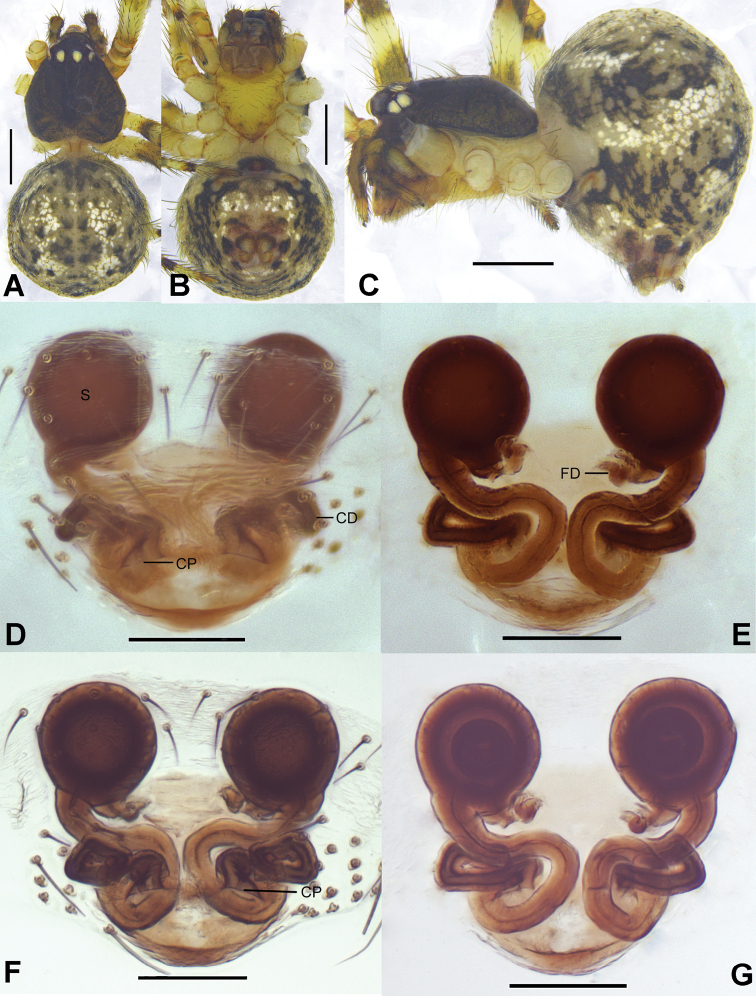
*Campanicolatauricornis* sp. nov. **A–C** female habitus (**A** dorsal view **B** ventral view **C** lateral view) **D–G** female epigynum (**D** ventral view, cleared **E** dorsal view, cleared **F** ventral view, cleared and embedded in Arabic gum **G** dorsal view, cleared and embedded in Arabic gum). Scale bars: 0.5 mm (**A–C**); 0.1 mm (**D–G**).

**Figure 11.**
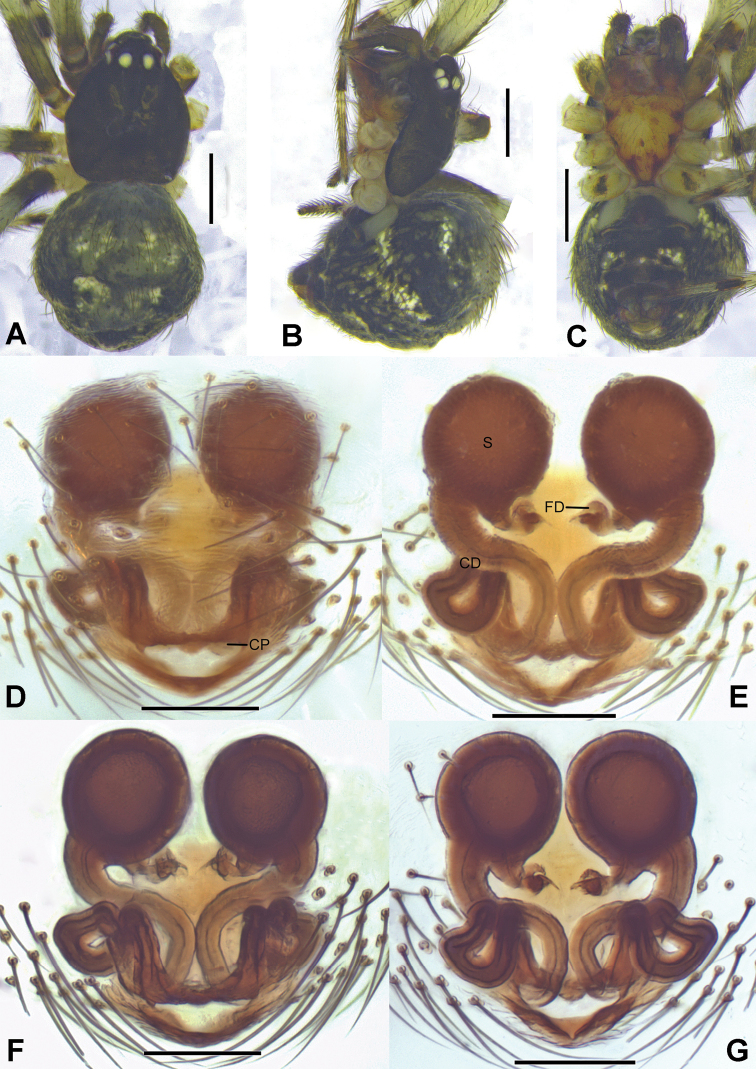
*Campanicolavolubilis* sp. nov. **A–C** female habitus (**A** dorsal view **B** lateral view **C** ventral view) **D–G** female epigynum (**D** ventral view, cleared **E** dorsal view, cleared **F** ventral view, cleared and embedded in Arabic gum **G** dorsal view, cleared and embedded in Arabic gum). Scale bars: 0.5 mm (**A–C**); 0.1 mm (**D–G**).

This new species is similar to *C.volubilis* sp. nov. in having the spherical spermathecae, the long and looped copulatory ducts which start from the lateral and anterior part of the atrium, extend anteriorly, then curve several times and enter the spermathecae laterally and ventrally, but can be distinguished from the latter by the following characteristics: 1. The copulatory pores are slightly separated from each other in this new species (Fig. 10D, F), but close to each other in *C.volubilis* sp. nov. (Fig. 11D, F); 2. The spermathecae are widely separated in this new species (Fig. 10D–G), but slightly separated in *C.volubilis* sp. nov. (Fig. 11D–G).

##### Etymology.

The specific name is derived from the Latin adjective *tauricornis*, meaning ox horn-shaped, referring to the shape of the copulatory ducts in dorsal view; adjective.

##### Description.

**Male** unknown. **Female (holotype)**: Total length 4.02. Prosoma 1.59 long, 1.52 wide, brownish black. Sternum 0.86 long, 0.95 wide, yellow, with sparse setae. Opisthosoma 2.18 long, 2.16 wide, oval, mottled, armed with sparse black setae, both dorsum and venter with irregular black plaque and white spots. Anal tubercle yellowish. Spinnerets brownish, without colulus (Fig. 10A–C). Diameters of eyes: AME 0.09, ALE 0.07, PME 0.10, PLE 0.08. Interdistances of eyes: AME-AME 0.08, AME-ALE 0.03, PME-PME 0.07, PME-PLE 0.05. Clypeus 0.19 high, brown. Chelicerae yellow, promargin with 2 teeth. Endites brown. Labium 0.24 long, 0.08 wide, yellowish-brown, rectangular, fused with sternum. Measurements of legs: I 4.78 (1.54, 0.34, 1.00, 1.20, 0.70), II 3.32 (1.10, 0.32, 0.64, 0.76, 0.50), III 2.48 (0.84, 0.21, 0.48, 0.55, 0.40), IV 3.711 (1.27, 0.34, 0.75, 0.83, 0.53). Leg formula: I-IV-II-III. Legs yellow to brown, with brownish black ring pattern. Epigynum: atrium depression and oblate; copulatory pores distinguishable, located on anterior margin of atrium; copulatory ducts long, winding complexly, the part of copulatory ducts near copulatory pores is evidently more sclerotized than the part near spermathecae, connected with spermathecae from posterior part; spermathecae spherical, spacing obvious; fertilization ducts short, curved and tapering (Fig. 10D, E).

##### Distribution.

China (Hainan) (Fig. 16).

#### Campanicola volubilis

Taxon classificationAnimaliaAraneaeTheridiidae

Li & Liu sp. nov.

25CFE359-E2BC-5C8C-8BF7-217961E2C376

http://zoobank.org/93EBE9D7-723A-4A20-B7AF-7B24D165F85D

Figs 11 , 15 , 16

##### Type material.

***Holotype*: ♀, China, Yunnan Province**: Xishuangbanna Dai Autonomous Prefecture, Menghai county, Bulang Nationality Township (21°34' 37"N, 100°20'24"E, 1130 m), 29 July 2020, Z.W. Deng, W. Zhang, Y.T. Zhang, R. Zhong and Z.C. Li leg. **Paratypes**: 2 ♀, same data as holotype.

##### Diagnosis.

See the diagnosis under *C.tauricornis* sp. nov.

##### Etymology.

The specific name is derived from the Latin adjective *volubilis*, meaning winding, referring to the shape of the part of the copulatory ducts near the copulatory pores in ventral view; adjective.

##### Description.

**Male** unknown. **Female (holotype)**: Total length 3.74. Prosoma 1.74 long, 1.44 wide, brownish black. Sternum 0.99 long, 0.88 wide, yellow, some reddish-brown patches on the margin, with sparse setae. Opisthosoma 2.02 long, 1.96 wide, oval, brownish black, with black setae, both dorsum and venter with white spots. Anal tubercle and spinnerets brownish black, without colulus (Fig. 11A–C). Diameters of eyes: AME 0.08, ALE 0.07, PME 0.10, PLE 0.07. Interdistances of eyes: AME-AME 0.08, AME-ALE 0.02, PME-PME 0.09, PME-PLE 0.05. Clypeus 0.20 high, brownish black. Chelicerae brown to yellow from base to end, promargin with 2 teeth. Endites yellow-brown. Labium 0.21 long, 0.06 wide, brown, rectangular, fused with sternum. Measurements of legs: I 5.05 (1.63, 0.35, 1.03, 1.38, 0.66), II 3.19 (1.01, 0.25, 0.64, 0.78, 0.51), III 2.48 (0.79, 0.27, 0.45, 0.54, 0.43), IV 3.82 (1.31, 0.34, 0.79, 0.87, 0.51). Leg formula: I-IV-II-III. Legs yellowish, with brownish black ring pattern. Epigynum: atrium depression and oblate; copulatory pores big and distinguishable, located on anterior margin of atrium; copulatory ducts long, winding complexly, the part of copulatory ducts near copulatory pores is evidently more sclerotized than the part near spermathecae, connected with spermathecae from posterior part; spermathecae spherical; fertilization ducts short, curved and tapering (Fig. 11D, E).

##### Distribution.

China (Yunnan) (Fig. 16).

#### Genus *Parasteatoda* Archer, 1946

##### Parasteatoda ducta

Taxon classificationAnimaliaAraneaeTheridiidae

(Zhu, 1998)

847B5C9E-93C1-5D62-A105-3D0D7979C58A

**Figure 12.**
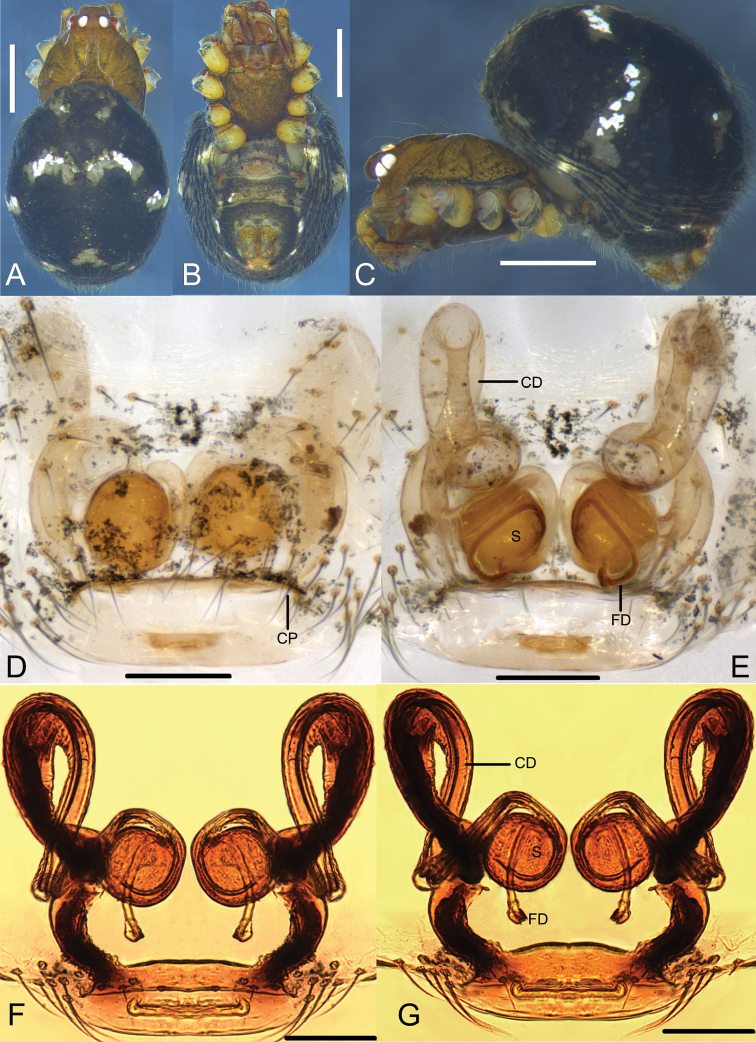
*Parasteatodaducta* (Zhu, 1998) **A–C** female habitus (**A** dorsal view **B** ventral view **C** lateral view) **D–G** female epigynum (**D** ventral view, cleared **E** dorsal view, cleared **F** ventral view, cleared and embedded in Arabic gum **G** dorsal view, cleared and embedded in Arabic gum). Scale bars: 0.5 mm (**A–C**); 0.1 mm (**D–G**).

**Figure 13.**
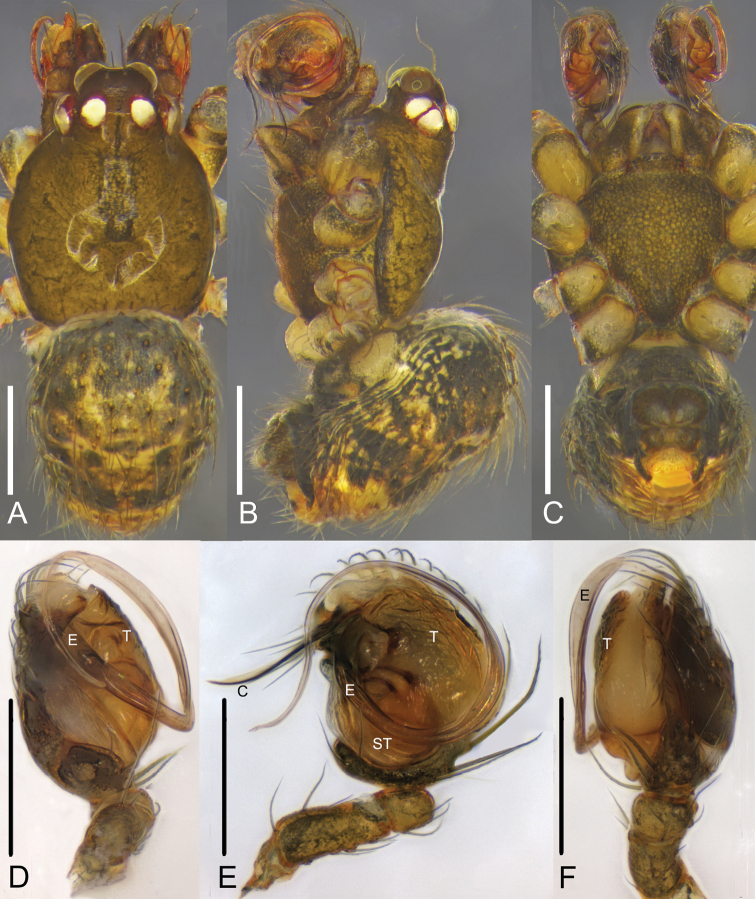
*Parasteatodaducta* (Zhu, 1998) **A–C** male habitus (**A** dorsal view **B** lateral view **C** ventral view) **D–F** male left palp (**D** prolateral view **E** ventral view **F** retrolateral view). Scale bars: 0.5 mm (**A–C**); 0.1 mm (**D–F**).

**Figure 14.**
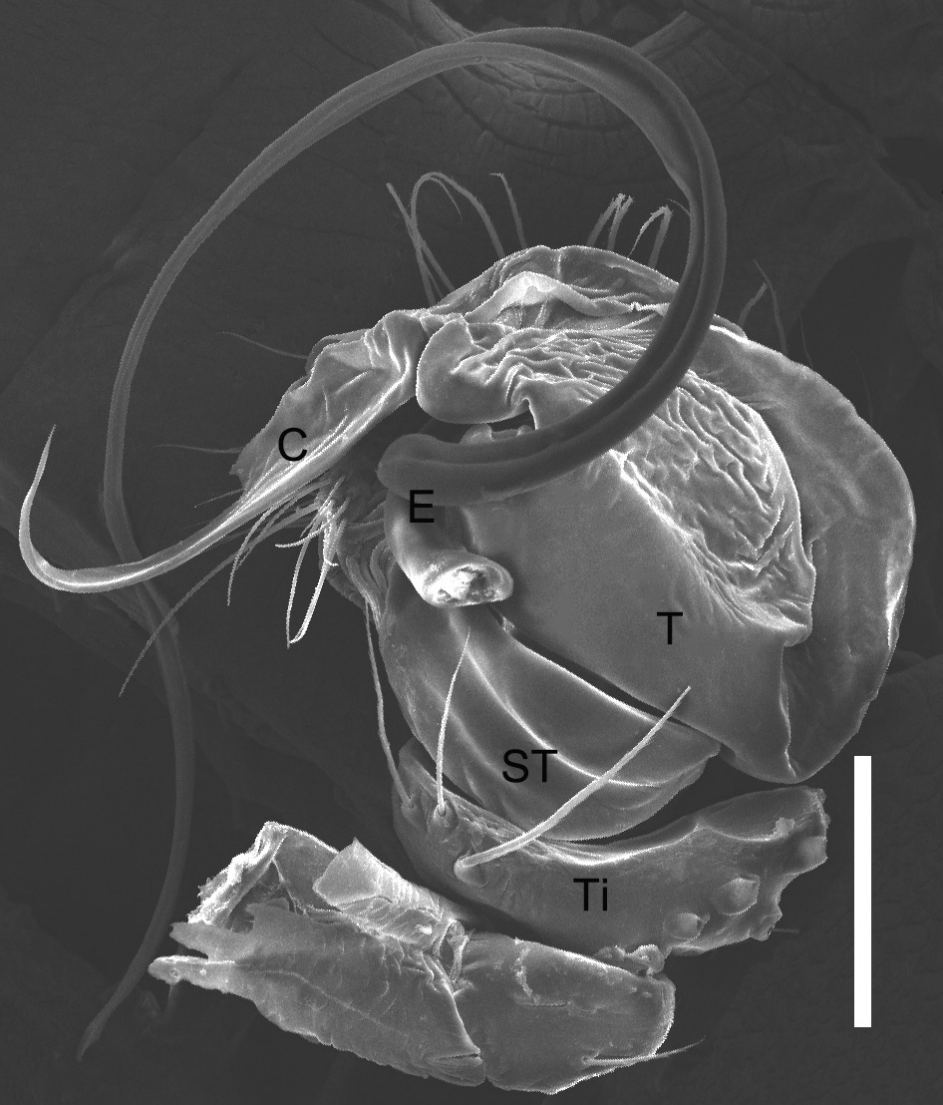
*Parasteatodaducta* (Zhu, 1998) male left palp (ventral view, embolus broken). Imaged by field emission scanning electron microscope: FE-SEM JSM7100F, JEOL, JP. Scale bars: 0.1 mm.

**Figure 15.**
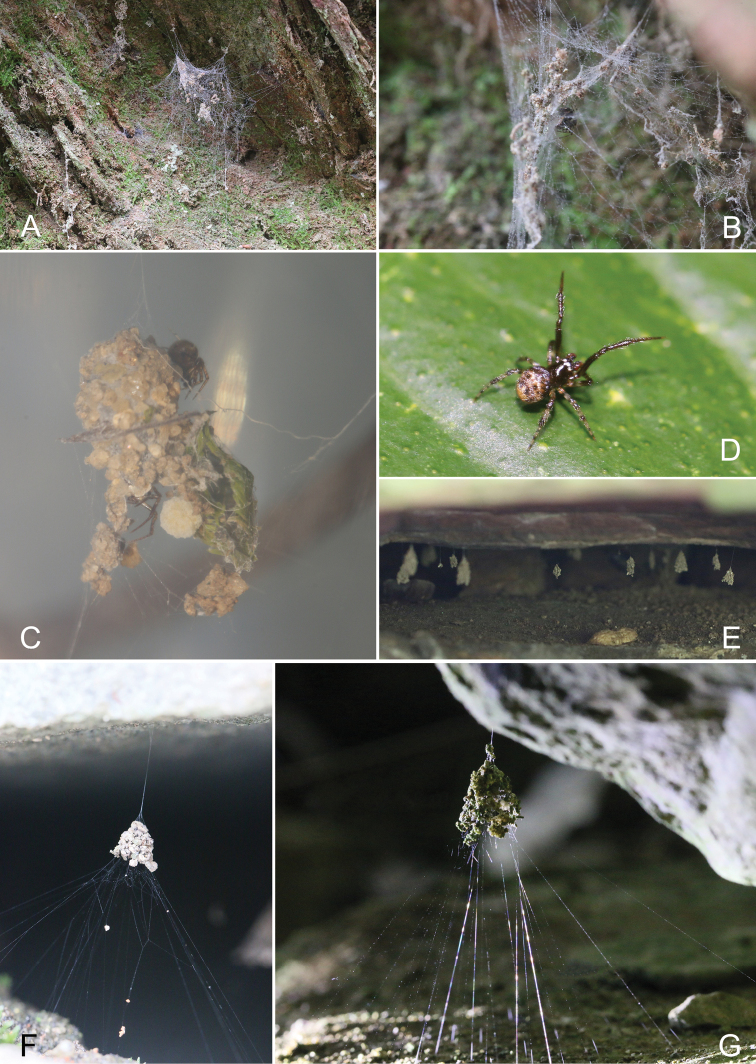
Field photographs **A, B***C.volubilis* sp. nov. (**A** web and bell-shaped retreat **B** female in the web **C, D***P.ducta* (**C** male outside retreat, female with egg sac in retreat **D** male on leaf) **E** retreat of *C.ferrumequina***F, G** retreat of *C.campanulata*. Photograph by Zichang Li.

**Figure 16.**
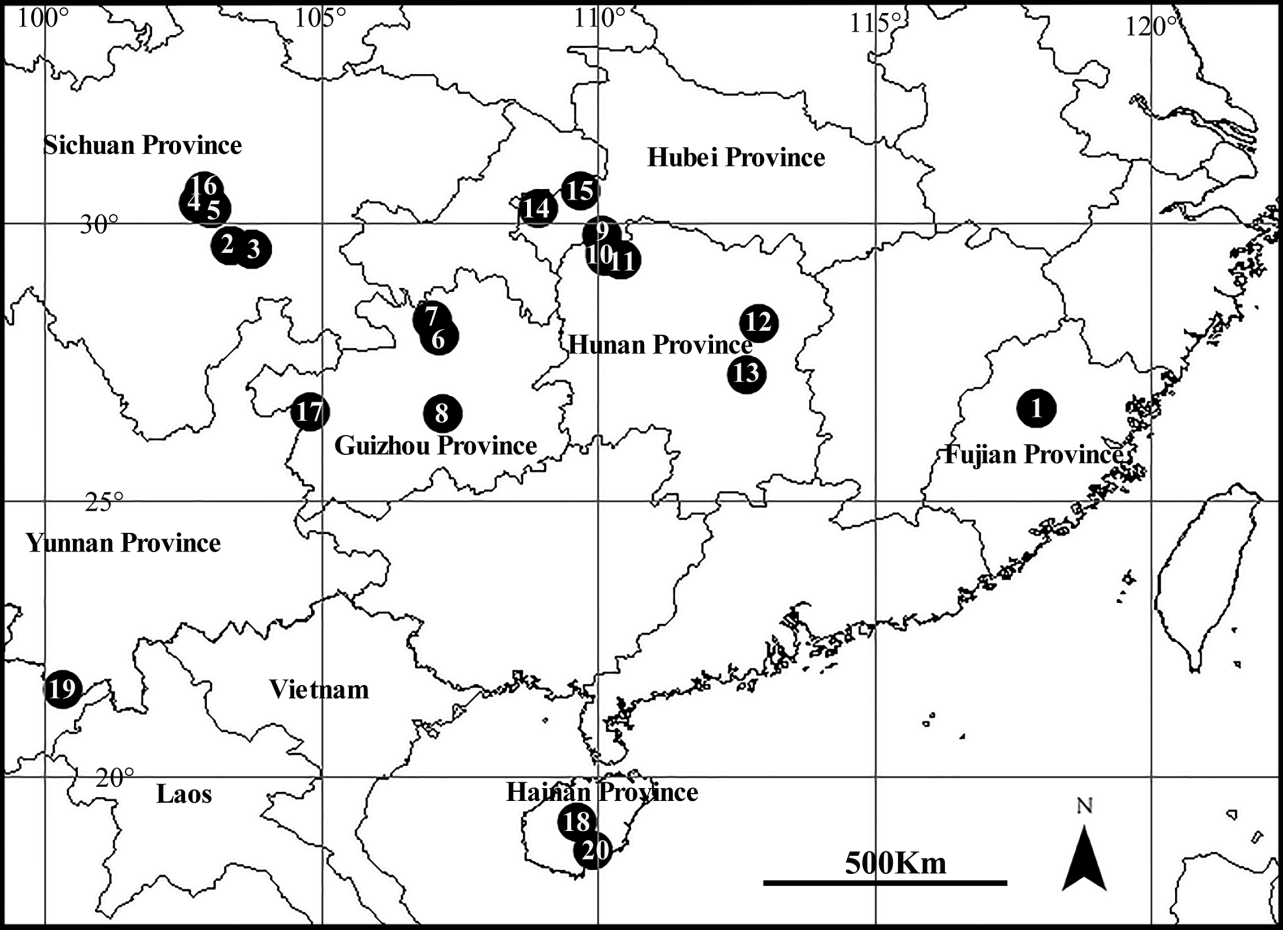
Collection localities. *C.anguilliformis* sp. nov. (**1)**; *C.campanulata* (**2–15)**; *C.falciformis* sp. nov. (**2–5, 16)**; *C.ferrumequina* (**1, 11)**; *C.heteroidea* sp. nov. (**17)**; *C.tauricornis* sp. nov. (**18)**; *C.volubilis* sp. nov. (**19)**; *P.ducta* (**20)**.

Figs 12 , 13 , 14 , 15 , 16

Achaearanea ducta Zhu, 1998: 107, f. 64A–C (description of female); Song, Zhu and Chen 1999: 90, f. 39E, F (female).
Parasteatoda ducta : Yoshida 2008: 39 (female transferred from Achaearanea).

###### Material examined.

**China, Hainan Province**: 2 ♂, 10 ♀, Lingshui County, Diaoluoshan National Forest Park (18°40'02"N, 109°55'26"E, 80 m), 24 April 2019, F.X. Liu, J. Liu and F.J. Liu leg.

###### Diagnosis.

Males of *P.ducta* are similar to *Parasteatodacingulata* (Zhu, 1998) and *Parasteatodatransipora* (Zhu & Zhang, 1992) in having a long and looped embolus (Figs 13D–F, 14), but can be distinguished from them by the long and slender conductor with a sharp end (Figs 13D, E, 14). Females of *P.ducta* differ from all other *Parasteatoda* species by the relatively smaller spermathecae enclosed laterally by the copulatory ducts (Fig. 12D, E).

###### Description.

**Male**: Total length 1.25. Prosoma 0.71 long, 0.56 wide, brownish yellow, with dark brown margin. Sternum 0.41 long, 0.36 wide, yellow, peltate. Opisthosoma 0.59 long, 0.48 wide, oval, dorsum yellow to brown, with long setae and black patches, venter black. Anal tubercle yellow. Spinnerets brown, without colulus (Fig. 13A–C). Diameters of eyes: AME 0.09, ALE 0.07, PME 0.07, PLE 0.06. Interdistances of eyes: AME-AME 0.06, AME-ALE 0.03, PME-PME 0.05, PME-PLE 0.04. Clypeus 0.14 high, dark brown. Chelicerae tawny, promargin with 2 teeth. Endites yellow to dark brown. Labium 0.15 long, 0.04 wide, yellow, rectangular, fused with sternum. Measurements of legs: I 2.43 (0.75, 0.21, 0.56, 0.51, 0.40), II 1.77 (0.53, 0.20, 0.37, 0.35, 0.32), III 1.395 (0.44, 0.17, 0.26, 0.26, 0.26), IV 1.79 (0.56, 0.20, 0.37, 0.34, 0.32). Leg formula: I-IV-II-III. Femur white to light yellow, patella, tibia, tarsus and metatarsus yellow, with dark brown ring pattern. Palp: yellowish-brown, tibia scoop-shaped, with long setae at both sides; cymbium small, brownish black, with a row of neat, thick, curved and long setae on the anterior margin; embolus long and bends more than one round, middle part flat, distal part thin; conductor sclerotized and tapering; tegulum large, subtegulum small, both with a groove for embolus (Figs 13D–F, 14).

**Female**: Total length 2.04. Prosoma 0.92 long, 0.78 wide, brownish yellow, with dark brown margin. Sternum 0.49 long, 0.47 wide, yellow and peltate, with sparse setae. Opisthosoma 1.47 long, 1.18 wide, oval, dorsum brownish black, with a discontinuous wavy yellow transverse near the midpoint which contains sporadic white spots, venter brown. Anal tubercle and spinnerets yellow, surrounded with black ring, without colulus (Fig. 12A–C). Diameters of eyes: AME 0.07, ALE 0.07, PME 0.08, PLE 0.08. Interdistances of eyes: AME-AME 0.06, AME-ALE 0.03, PME-PME 0.08, PME-PLE 0.06. Clypeus 0.13 high, yellow. Chelicerae yellow, promargin with 2 teeth. Endites yellow. Labium 0.18 long, 0.06 wide, yellow, rectangular, fused with sternum. Measurements of legs: I 2.92 (0.92, 0.27, 0.60, 0.67, 0.46), II 2.11 (0.64, 0.25, 0.39, 0.45, 0.38), III 1.78 (0.53, 0.23, 0.32, 0.37, 0.33), IV 2.68 (0.83, 0.29, 0.55, 0.59, 0.42). Leg formula: I-IV-II-III. Femur white to light yellow, patella, tibia, metatarsus and tarsus orange yellow with dark brown ring. Epigynum: atrium big, depression and oval, its width is only a little narrower than epigynum’s; copulatory pores located on the anterior margin of atrium; copulatory ducts long, winding, surround spermathecae, and connected with spermathecae from the posterior part; spermathecae small, spherical; fertilization ducts long and thin (Fig. 12D–G).

###### Distribution.

China (Hainan) (Fig. 16).

## Discussion

Eight cobweb spider species building detritus-based, bell-shaped retreats from China are reported in the current paper, including five new *Campanicola* species, two known *Campanicola* species and one known *Parasteatoda* species. However, all new species are reported only based on the female specimens because the male individuals are relatively difficult to collected in the field. According to our experience both in field collection and feeding in the lab, the males after maturity often stop weaving webs and leave their retreats to search for females. We investigated more than 500 retreats of *C.campanulata* in the field for research focused on its web-weaving behaviour, and only 21 males were collected, often found together with females (manuscript in preparation). Therefore, more thorough collecting or rearing juveniles in the lab may be needed to find further male individuals for these new species in the future.

The type of bell-shaped retreat is rare in the family Theridiidae, and found only in four related genera: *Achaearanea*, *Cryptachaea*, *Parasteatoda* and *Campanicola* (Henschel and Jocqué 1994; Zhu 1998; Yoshida 2009, 2015, 2016). All of them belong to the subfamily Theridiinae according to both morphological and molecular phylogeny (Agnarsson 2004; Liu et al. 2016). The relationship between these four genera is relatively complicated. And their respective monophyly remains to be verified. Many species in *Parasteatoda* and *Cryptachaea* were transferred from *Achaearanea*, and some species in *Parasteatoda* were transferred to *Campanicola* (Yoshida 2008; Backup et al. 2010; Yoshida 2015). Moreover, some *Parasteatoda* species are quite different from others, for example, the spermathecae of *Parasteatodatransipora* (Zhu & Zhang, 1992) and *Parasteatodacingulata* (Zhu, 1998) is significantly smaller than other *Parasteatoda* species’ (Zhu 1998). In addition, information on web-weaving behaviour (especially focused on the detritus-based, bell-shaped retreat) and ecology (such as the trade-off between the safety and predation) of these spiders is still limited. Therefore, a natural next step upon completing this taxonomic study would be to analyse and understand the evolution of the retreat and related traits.

## Supplementary Material

XML Treatment for Campanicola

XML Treatment for Campanicola campanulata

XML Treatment for Campanicola ferrumequina

XML Treatment for Campanicola anguilliformis

XML Treatment for Campanicola falciformis

XML Treatment for Campanicola heteroidea

XML Treatment for Campanicola tauricornis

XML Treatment for Campanicola volubilis

XML Treatment for Parasteatoda ducta
